# Joint representation learning for oncology applications

**DOI:** 10.1093/bioinformatics/btaf597

**Published:** 2025-10-29

**Authors:** Tanya Nandan, Bowen Fan, Samuel Håkansson, Catherine R Jutzeler, Sarah C Brüningk

**Affiliations:** Department of Biosystems Sciences and Engineering (D BSSE), ETH Zürich, Zürich 8092, Switzerland; Department of Health Sciences and Technology (D HEST), ETH Zürich, Zürich 8092, Switzerland; Department of Quantitative Medicine, University of Zürich, Zürich 8006, Switzerland; Department of Medical Oncology & Haematology, University Hospital Zürich, Zürich 8091, Switzerland; Department of Health Sciences and Technology (D HEST), ETH Zürich, Zürich 8092, Switzerland; Department of Health Sciences and Technology (D HEST), ETH Zürich, Zürich 8092, Switzerland; Department of Radiation Oncology, Inselspital, Bern University Hospital and University of Bern, Bern 3012, Switzerland

## Abstract

**Motivation:**

The integration of tumour imaging data and molecular sequencing information can advance our understanding of cancer biology by combining complementary perspectives of tumour phenotype and genotype. However, integrating multi-modal data across heterogeneous and high-dimensional data domains remains a significant computational challenge.

**Results:**

Here, we introduce an unsupervised manifold alignment approach for real-world data integration based on Joint Multidimensional Scaling (Joint MDS) and extend it to a three-modality framework (Joint MDS3). We apply this method to integrate radiomic features from magnetic resonance imaging (MRI) with transcriptomic, epigenomic, and copy number variation (CNV) data from patients with glioblastoma multiforme (GBM) and lower-grade gliomas (LGG). Compared to baselines such as Pamona and single-cell optimal transport (SCOTv2), Joint MDS consistently outperforms baseline Pamona in cases and achieves competitive performance relative to baseline SCOTv2, outperforming its fraction of samples closer to an incorrect match (FOSCTTM) in four out of six cases. Joint MDS attains an average label transfer accuracy of 74.8%, approximately 4% higher than that of Pamona and SCOTv2, and reduces FOSCTTM to 51% or less across real-world datasets. We further demonstrate our extension JointMDS3 on both synthetic and real-world examples. Our results highlight the potential of Joint MDS to enhance the integration of diverse data types into a unified representation, ultimately advancing computational approaches in complex diseases.

**Availability and implementation:**

The implementation of our work is available at gitlab.ethz.ch/BMDSlab/publications/oncology/joint-representation-learning-for-oncology-applications and archived at doi.org/10.5281/zenodo.17219404

## 1 Introduction

Cancer, characterized by its heterogeneity and dynamic evolution in response to microenvironmental cues, presents significant challenges for therapeutic interventions (Bozic and Wu 2020). The diverse molecular landscape and adaptive capabilities of cancer cells often lead to treatment resistance, necessitating in-depth molecular characterization of tumours to improve patient outcomes. Different data modalities capture different aspects of an individual tumour, and advances in sequencing technologies have revolutionized our ability to understand the molecular intricacies of tumours. While magnetic resonance imaging scans (MRIs) allow for detailed visualization of the entire tumour, molecular sequencing data, such as transcriptomics or DNA methylation patterns, offer molecular-level insight into gene expression patterns. However, they are typically limited to the sampled region (biopsy), potentially introducing biases. Existing literature predominantly explores the correlation between imaging phenotypes and genomic data, a field known as radiogenomics (Chaddad *et al.* 2019, Liu *et al.* 2023). Integrating multiple data modalities offers the potential to harness complementary strengths to gain a more comprehensive understanding of the phenotypic landscape (Boehm *et al.* 2022). A prime example is integrating molecular sequencing data with imaging features, such as radiomics (Lambin *et al.* 2012).

Unsupervised manifold alignment is a computational technique to integrate data from multiple modalities in a way that preserves the underlying structure and relationships between the data points (Wang and Mahadevan 2009, Cui *et al.* 2014). Optimal transport has emerged as a powerful technique for manifold alignment, aiming to map one dataset’s probability distribution to another’s by minimizing the transportation cost while maintaining the structural integrity of the data (Cuturi 2013, Peyré and Cuturi 2019). Classical optimal transport algorithms take the data instances as input and assumes the two data distributions originate from the same (feature) space, making the alignment of dissimilar ‘domains’ challenging (Peyré and Cuturi 2019). This limitation is overcome by the Gromov–Wasserstein extension, which uses pairwise intra-domain dissimilarity matrices as input instead of the data instances (Memoli 2011). Manifold alignment methods, such as Pamona and single-cell optimal transport (SCOTv2), have been used to integrate and align multi-omics data (Cao *et al.* 2021, Demetci *et al.* 2022). Pamona employs geodesic distance matching to reconcile differences between heterogeneous datasets, while SCOTv2 applies optimal transport theory to align distributions across modalities. Despite the advancements achieved by these two methods, handling complex biological data remains challenging, particularly in scenarios with high dimensionality (Chen *et al.* 2023). Joint Multidimensional Scaling (Joint MDS) uses the Gromov–Wasserstein extension of optimal transport with classical MDS to simultaneously align and embed data from two domains into a common low-dimensional space in the absence of explicit correspondences between the two data modalities (Chen *et al.* 2023). However, like many manifold alignment algorithms, Joint MDS is limited to two input domains; using optimal transport to align more than two domains is an active field of research (Pass 2015, García Trillos and Jacobs 2023). Also, Joint MDS has only been tested on aligning data from similar biological domains, such as gene expression and chromatin accessibility data (Chen *et al.* 2023).

In this study, we investigate the application of Joint MDS for brain tumour characterization. Brain tumours account for 90% of cancers affecting the central nervous system (Cancer.Net 2023) and impact critical functions such as motor skills and cognition (Ilic and Ilic 2023). Gliomas, originating in glial cells, represent 80% of malignant brain tumours (Wang *et al.* 2022) and are classified into subtypes based on histological and molecular characteristics, with malignancy grades ranging from WHO grade I to IV (Weller *et al.* 2015). We focus on lower grade gliomas (LGG), classified as grade I-II, and glioblastoma multiforme (GBM), classified as grade IV, that are typically diagnosed using MRIs. LGGs are slow-growing tumours with defined borders, whereas GBMs are aggressive, fast-growing masses with irregular borders (Malik *et al.* 2021). This study has two primary objectives. First, we explore the novel application of Joint MDS on real-world healthcare data by applying it to dissimilar domain pairs, such as MRI and molecular sequencing data, to learn clinically meaningful joint representations through a multi-modal approach. Second, we extend the Joint MDS algorithm to support the alignment of three similar domains for enhanced multi-modal integration. Although diagnostic labels like GBM and LGG exist, tumours exhibit continuous variation not captured by supervised methods. Using unsupervised Joint MDS to learn a shared representation space captures continuous variation better. We still use diagnostic labels for evaluation to show clinically relevant separation. However, Joint MDS also enables the discovery of novel subclusters beyond discrete classes, contributing to a more comprehensive understanding of the brain tumour landscape across data modalities.

## 2 Materials and methods

### 2.1 Algorithms

#### 2.1.1 Joint multidimensional scaling algorithm

Joint Multidimensional Scaling is an unsupervised manifold alignment method that effectively maps data from two domains to a common low-dimensional space. Joint MDS takes the pairwise dissimilarities of each data modality as the input, and the problem is formulated as a joint optimization task that alternates between two main steps: Multidimensional Scaling and Wasserstein-Procrustes Analysis (Borg and Groenen 2005, Alvarez-Melis *et al.* 2019).

Multidimensional Scaling is a dimensionality reduction method that preserves the pairwise dissimilarities between data instances (Borg and Groenen 2005). Given two matrices corresponding to the pairwise dissimilarities of two domains, $D\in R^{n\times n}\;\mathrm{and}\;D^{\prime }\in R^{n\prime \times n\prime }$, MDS minimizes the following stress function:


(1) $$\begin{array}{c} min\\ Z\in R^{n\times d},\;Z^{\prime }\in R^{n^{\prime }\times d} \end{array}\;\mathrm{stress}(Z,D,W)+\mathrm{stress}(Z^{\prime },D^{\prime },W^{\prime })$$

where W and $W^{\prime }$ are weights for the pairwise dissimilarities generally equal to $1/n^{2}$ and $1/n^{\prime 2}$, and $Z,Z^{\prime }$ correspond to the low-dimensional embeddings. The stress terms in (1) can be written out as:


(2) $$\begin{array}{c} min\\ Z\in R^{n\times d},\;Z^{\prime }\in R^{n^{\prime }\times d} \end{array}\sum_{i,j=1}^{n}w_{ij}{\left(d_{ij}-d(z_{i},z_{j})\right)}^{2}+\sum_{i\prime ,j\prime =1}^{n\prime }{w^{\prime }}_{i^{\prime }j^{\prime }}{\left({d^{\prime }}_{i^{\prime }j^{\prime }}\;-d(z_{i}^{\prime },z_{j}^{\prime })\right)}^{2}$$

The weighted MDS problem in (2) is solved using the SMACOF algorithm (de Leeuw 1977), which is based on the majorization theory. This approach minimizes a simpler surrogate function as a proxy for the original function. However, since the inputs to the Joint MDS algorithm come from two different domains, the resulting low-dimensional embeddings Z and $Z^{\prime }$ reside in separate, non-coherent subspaces without any known prior correspondences between the two spaces. Thus, embedding spaces need to be transformed and aligned to facilitate meaningful comparisons and joint visualization.

Wasserstein-Procrustes analysis finds correspondences between the two data instances to transform one of the two embeddings and then align them to a common low-dimensional subspace (Alvarez-Melis *et al.* 2019). The problem is formulated as:


(3) $$\begin{array}{c} min\\ O\in O_{d},\;P\in \Pi (a,b) \end{array}{〈P,d^{2}(\mathrm{ZO},\;Z^{\prime })〉}_{F}-\varepsilon H(P)$$

This involves finding a minimum cost matrix, $P\;\in R^{n\times n^{\prime }}$, to transform one probability distribution into another, and orthogonal transformation function $O\in R^{d}$. ε corresponds to the entropic regularization term that makes the objective strongly convex to solve for P more easily (Grave et al. 2019). Wasserstein-Procrustes is carried out through an alternating optimization scheme to iteratively update P and O, during which embeddings Z and $Z^{\prime }$ are fixed. First, fixing O yields the classical discrete optimal transport problem with entropic regularization, ε, for which Sinkhorn’s algorithm is used to efficiently solve for P (Cuturi 2013, Peyré and Cuturi 2019). Next, fixing P yields the classic orthogonal Procrustes problem, and singular value decomposition of $Z^{T}PZ^{\prime }$ solves for O (Alvarez-Melis *et al.* 2019, Bai and Bartoli 2022).

The overall Joint MDS problem is then formulated as:


(4) $$\begin{array}{c} min\\ Z\in R^{n\times d},\;Z^{\prime }\in R^{n^{\prime }\times d},\;\\ O\in O_{d},\;P\in \Pi (a,b) \end{array}\;\mathrm{stress}(Z,D,W)+\mathrm{stress}(Z^{\prime },\;D^{\prime },\;W^{\prime })+2\lambda {〈P,d^{2}(\mathrm{ZO},\;Z^{\prime })〉}_{F}$$

The first two stress terms capture the distance deviation between the low-dimensional embeddings and the original pairwise dissimilarities for each domain, and the last term quantifies correspondences between the instances of the two domains with a matching penalty parameter λ (Chen *et al.* 2023). Overall, the Joint MDS problem in (4) is solved by first randomly initializing the two low-dimensional embeddings using the SMACOF algorithm. Then, the algorithm alternates between solving the Wasserstein-Procrustes problem to update P and O, and using the updated P and O to solve the weighted MDS problem to update embeddings Z and $Z^{\prime }$. The algorithm iterates over these two main steps until convergence, i.e. minimal change in the transportation cost matrix P.

#### 2.1.2 Extending joint MDS to align three domains

**Figure 1.**
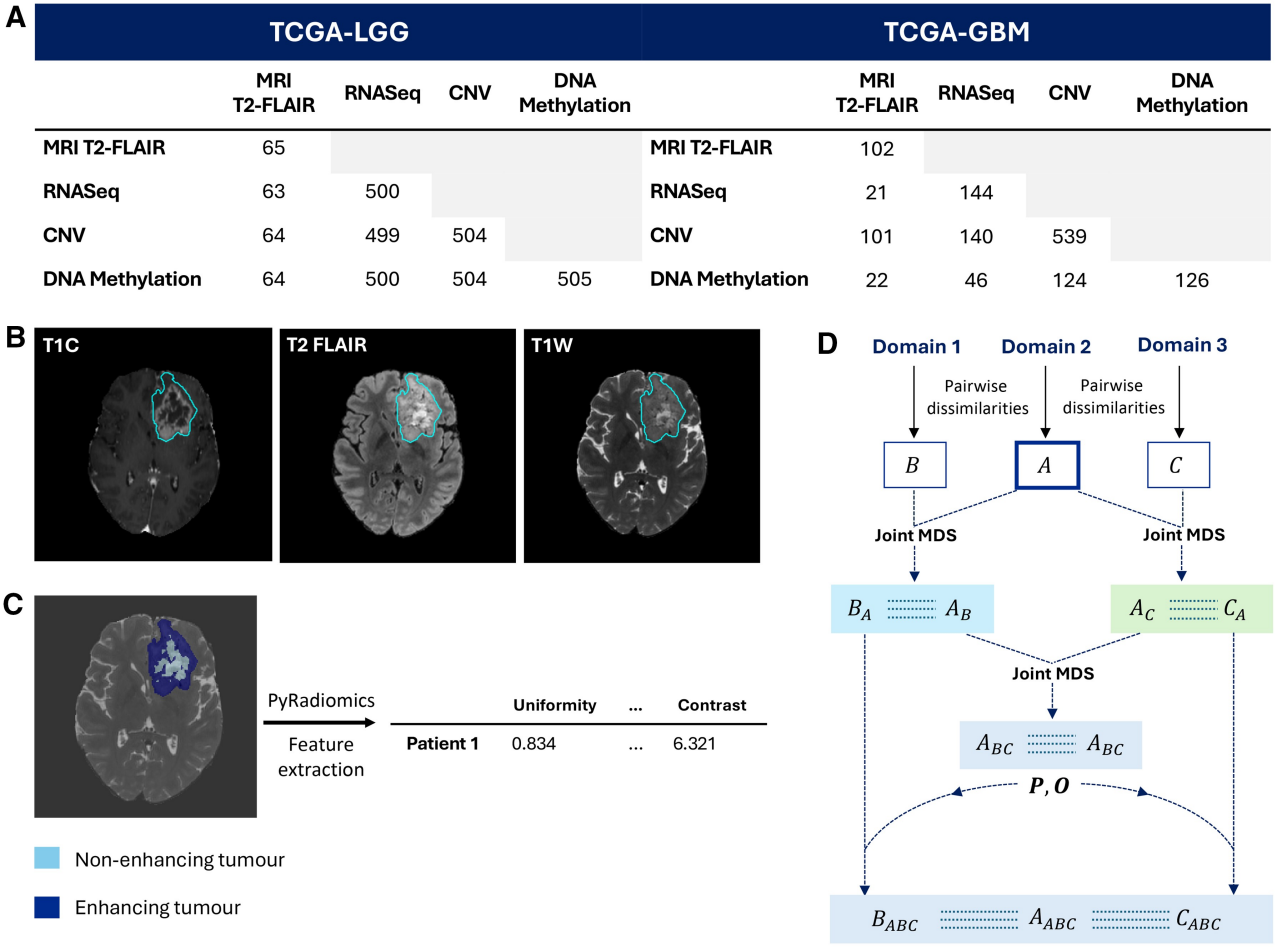
(A)The number of shared patients across combinations of sequencing and imaging data modalities. (B) 2D slice of three different MRI contrast volumes containing the largest tumour area for one patient. (C) 2D MRI T2-FLAIR contrast slice showing the non-enhancing tumour region that has undergone extensive cell death, and the enhancing tumour region where tumour cells are actively dividing. PyRadiomics package is employed to extract features from the non-enhancing and enhancing tumour region. (D) Schematic of the Joint MDS3 to three domains, where each a low-dimensional subspace is coloured differently.

We extended the Joint MDS algorithm to align and transform datasets from three domains (Joint MDS3), enhancing multi-modal data integration capabilities. In this extension, we assume one of three datasets—A, B, or C—as the core dataset, typically identified as the most informative based on prior literature. We then perform three successive rounds of pairwise Joint MDS. Selecting A as the core dataset, we align B and C to dataset A, resulting in low-dimensional embeddings B_A_ and C_A_, alongside two embeddings for A: A_B_ and A_C_. Subsequently, we perform Joint MDS on the low-dimensional embeddings A_B_ and A_C_ to find a common subspace that is now aligned to all three domains, giving us A_BC_. Finally, we use the transportation cost matrix P and orthogonal transformation function O from aligning A_B_ and A_C_ to align and transform B_A_ and C_A_, obtaining B_AC_ and C_AB_. In this way, we obtain low-dimensional embeddings A_BC_, B_AC_, and C_AB_ that reside in a common subspace with respect to all three domains. The overall approach is summarized in Algorithm 1 (Fig. 1D).

Algorithm 1 Joint MDS3

1. **Input**: distance matrices $D_{1},D_{2},\;D_{3}\in R^{n\times n}$, matching penalty λ and entropic regularization ϵ

2.  Set $D_{1}$ as core

3. Align Domain 2 and Domain 3 to Domain 1

  a  $\mathrm{Joint}\;\mathrm{MDS}(D_{1},\;D_{2})$ to get $Z_{1},\;Z_{2}\in R^{n\times d}$

  b  $\mathrm{Joint}\;\mathrm{MDS}(D_{1},\;D_{3})$ to get $Z_{1}^{\prime },Z_{3}\in R^{n\times d}$

4.  Align low dimensional subspaces in step 3(a, b) by aligning Domain 1 low-dimensional embeddings, $Z_{1},\;Z_{1}^{\prime }\in R^{n\times d}$

  a.  $\mathrm{Joint}\;\mathrm{MDS}(Z_{1},\;Z_{1}^{\prime })$ to get transportation matrix P and transformation function O

5.  Align and transform $Z_{2},Z_{3}\;$using P and O from step 5

6. **Output**: low dimensional embeddings $Z_{1},\;Z_{2},\;Z_{3}\in R^{n\times d}$

### 2.2 Datasets and application

#### 2.2.1 Sequencing and imaging data acquisition

This project incorporates two principal data modalities: molecular sequencing and imaging data. Sequencing data was obtained from The Cancer Genome Atlas (TCGA) and MRIs were obtained from the 2023 Brain Tumour Segmentation Challenge (Bran Li *et al.* 2023). The sequencing category comprises 644 transcriptomic gene expression patient profiles (bulk RNA-seq Fragments Per Kilobase Million counts—FPKM), 631 DNA methylation profiles (Illumina Human Methylation 450 BeadChip array) and 1043 Copy Number Variants (CNV) profiles (focal score by gene). The imaging category consists of 1251 MRIs, of which 65 correspond to LGG and 102 correspond to GBM patients; the remaining 1084 patients’ labels were not publicly available. MRIs comprised of three contrasts (Fig. 1B): T1 contrast-enhanced (T1C), T2-weighted with fluid-attenuated inversion recovery (T2-FLAIR), and T2-weighted (T2W). Figure 1A shows the number of shared patients for different data modalities pairs.

#### 2.2.2 Data pre-processing: sequencing data modalities

The three different sequencing data modalities, RNA-seq, DNA methylation, and CNV reside in different high-dimensional spaces: RNA-seq data contains gene expression values for 60 484 genes, DNA methylation data consists of methylation values for 485 578 sites and CNV data consists of duplication or deletion events, encoded as integers (−1, 0, 1), for 19 730 genes. Pre-processing steps reduced the dataset dimensionality and selected the most meaningful features for further analysis. For each modality, features with zero variance were dropped. Next, statistical testing using the Mann–Whitney U-test followed by Bonferroni multiple testing correction was performed to select for features with significant difference (corrected *P*-values < .05) between LGG and GBM patients. Since the number of significant features exceeded 10 000, we selected the top 5000, 10 000, and 4000 most significant features (lowest corrected *P*-values) for RNA-seq, DNA methylation, and CNV, respectively. The processed datasets were visualized using t-distributed Stochastic Neighbour Embedding (t-SNE) (van der Maaten and Hinton 2008).

#### 2.2.3 MRIs: feature extraction and pre-processing

Radiomic features are quantitative descriptors extracted from medical images that capture the underlying patterns, shapes, and textures of tissues, providing a comprehensive characterization of tumour phenotypes. For this study, radiomic features were extracted from 3D MRI volumes using PyRadiomics (van Griethuysen *et al.* 2017), focusing on the tumour region, which includes non-enhancing tumour tissue and enhancing tumour tissue (Fig. 1C). Shape-based and texture features, which capture the size and intensity variations of connected regions, were specifically extracted using T2 FLAIR contrast. This process resulted in 1410 features that provide detailed information about the tumour’s morphology and heterogeneity. Due to incomplete label assignment between GBM and LGG patients, it was not possible to perform feature selection via statistical testing. Instead, features with a variance-to-mean ratio greater than 0.01 were kept, reducing the radiomic feature space dimensionality to 765 features. These features were visualized using t-SNE (van der Maaten and Hinton 2008).

#### 2.2.4 Synthetic datasets

In line with previous work (Chen *et al.* 2023), three synthetic datasets, namely Bifurcation, Swiss roll, and Circular frustum, were used to quantify the performance of the Joint MDS3. Pairs of each labelled synthetic dataset were obtained from (Liu *et al.* 2019), each containing 300 samples linearly projected to 1000- and 2000-dimensional feature spaces respectively, and injected with gaussian noise. A third domain for each of the synthetic datasets was generated by combining the two domains and computing their principal components to extract the most significant patterns and variations present. These principal components are then applied to the first of the two domains, followed by the addition of Gaussian noise, ϵ ∼ N(0,0.05), that introduces random variations following a normal distribution. This resulted in a third domain in a 500-dimensional feature space. Thus, for each synthetic dataset we have three domains, $D_{1}\in R^{1000},\;D_{2}\in R^{2000}$ and $D_{3}\in R^{500}$.

#### 2.2.5 Joint MDS applied to multi-modal data pairs

Joint MDS was applied to all pairwise combinations of the four data modalities: RNA-seq, DNA methylation, CNV and MRI T2-FLAIR radiomic features. The T2-FLAIR contrast was chosen as it enhances tumour abnormalities (Okuda *et al.* 1999) emphasizing its clinical relevance. The geodesic distance, which measures the shortest distance between two points along the surface of the data manifold, was used when computing the pairwise dissimilarity matrices for each modality since increased performance of Joint MDS was previously observed in this scenario (Chen *et al.* 2023). Computing this distance requires complete knowledge of the data manifold, which is often unavailable. This issue is circumvented by constructing a k-nearest neighbour graph to calculate the shortest path distances, which represent the sum of edge weights along the shortest path connecting two nodes in the graph. Next, an extensive grid search was performed to select the hyperparameters k, entropic regularization, ε, and matching penalty λ for each pair of modalities (see Supplementary Appendix S1).

The performance of Joint MDS is evaluated using two metrics: fraction of samples closer than the true match (FOSCTTM) and label transfer accuracy (Liu *et al.* 2019). FOSCTTM quantifies the proportion of samples from one dataset that are closer to samples from the other dataset instead of their true match. A lower FOSCTTM corresponds to a better alignment. The label transfer accuracy score corresponds to unsupervised heterogeneous domain adaptation (HDA), where a k-NN classifier (k = 5) is trained on one domain to estimate the labels of the second domain. For example, a classifier trained on patients’ transcriptomic features and labels tries to predict the labels of their radiomic features. It is similar to a classification accuracy score, where higher values are better. Joint MDS was run 50 times with different random seeds, and the mean ± standard deviation is reported. Joint MDS performance was compared to two baselines, Pamona and SCOTv2, implemented following the original work (Cao *et al.* 2021, Demetci *et al.* 2022).

#### 2.2.6 Validation and application of joint MDS3

Joint MDS3 was validated on the three (triplets) of synthetic datasets using the same hyperparameters as in the study from Cao *et al.* 2021. Then, Joint MDS3 was applied to two real-world datasets: (i) radiomic features of three different MRI contrast data, T1C, T2-FLAIR, and T2W and (ii) RNA-seq, DNA methylation, and CNV data modalities. FOSCTTM and accuracy scores were calculated for each pairwise application of Joint MDS (Algorithm 1) and their averages are reported as the overall performance scores.

## 3 Results

### 3.1 Dataset exploration—sequencing, imaging and synthetic

**Figure 2.**
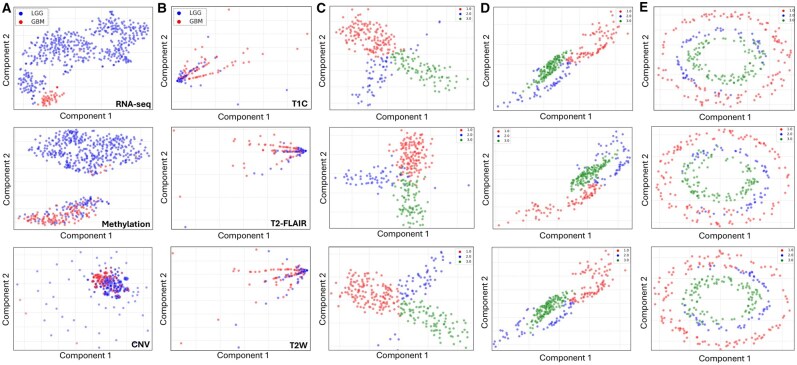
(A) Pre-processed sequencing of RNA-seq (top), DNA methylation (middle), and CNV (bottom) data. (B) Pre-processed imaging data of MRI contrasts T1C (top), T2-FLAIR (middle) and T2-weighted (bottom). GBM patients are coloured in red and LGG patients in blue. (C–E) Synthetic dataset triplets Bifurcation (C), Swiss roll (D), and Circular Frustrum (E) after dimensionality reduction coloured by their labels.

The sequencing and radiomic features visualized using t-distributed Stochastic Neighbour Embedding (t-SNE) (van der Maaten and Hinton 2008) revealed underlying patterns present within each modality (Fig. 2). RNA-seq and DNA methylation data (Fig. 2A) demonstrated clear segregation of GBM and LGG patients. Despite the class imbalance in these two modalities, with 82% of patients diagnosed as having LGG tumours, the separation of the GBM patients’ cluster was still discernible and not overshadowed by the dominant class. CNV data exhibited a less distinct separation of GBM and LGG patients, marked by circular clustering artefacts possibly attributed to the ordinal nature of the CNV values (−1, 0, 1). An interesting result from the Mann–Whitney U tests to identify significant features in RNA-seq data was a novel transcript antisense to the TTC39C gene (*P* < 0.001). This transcript is involved in anaphase regulation and has higher expression in GBM patients than LGG patients, suggesting its potential involvement in GBM tumour aggressiveness and progression (Protein Atlas 2022). Differentiating between GBM and LGG patients based on the extracted radiomic features from MRIs is unclear, with GBM patients exhibiting a more dispersed distribution in all three contrasts (Fig. 2B). In the synthetic data label distribution in the Bifurcation, Swiss roll, and Circular Frustrum show a clear separation between the three labels 1, 2, and 3 (Fig. 2C–E).

### 3.2 Joint MDS application on multi-modal data pairs

**Figure 3.**
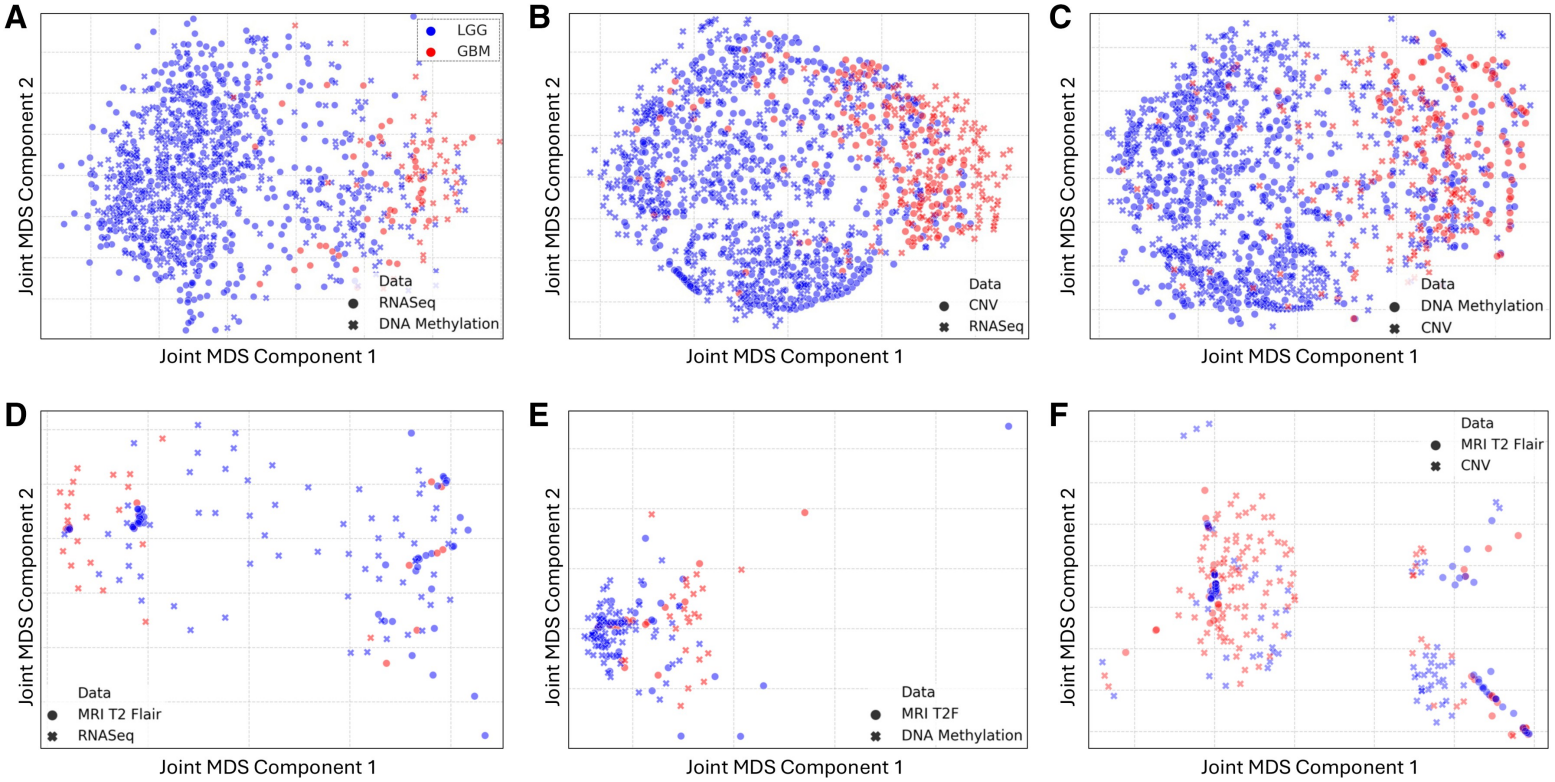
Joint visualization of the results from performing Joint MDS on different pairwise combinations of sequencing and imaging data modalities. Top row (A–C) corresponds to different pairwise combinations of sequencing modalities and the bottom row corresponds (D–F) to combination of one sequencing modality with the MRI T2-FLAIR imaging modality. GBM patients are in red and LGG patients in blue.

Joint MDS was performed on pairwise combinations of the pre-processed sequencing and radiomic features with hyperparameters selected through parameter grid search. Figure 3 shows the resulting joint visualization plots and Table 1 summarizes the Joint MDS performance as compared to two baselines, SCOTv2 and Pamona (see Supplementary Appendix S2.3 for more evaluation results).

**Table 1.** Results of Joint MDS performance on different modality pairs.^a^

Modality	Method	FOSCTTM	*P*-value	Transfer accuracy	*P*-value
RNAseq + Methylation	Joint MDS	27.5 ± 0.02	–	90.9 ± 0.004	–
	Baseline—Pamona	30.5	1.6e–29	93.3	7.7e–43
	Baseline—SCOTv2	29.0	3.7e–5	90.0	1.5e–22
RNAseq + CNV	Joint MDS	**43.5 **± 0.01	–	**81.2 **± 0.01	–
	Baseline—Pamona	52.9	3.8e–56	58.7	3.5e–56
	Baseline—SCOTv2	45.1	7.8e–26	79.8	6.0e–14
CNV + Methylation	Joint MDS	41.5 ± 0.04	–	77.7 ± 0.02	–
	Baseline—Pamona	52.8	1.8e–34	63.5	4.1e–9
	Baseline—SCOTv2	37.4	2.8e–10	80.1	2.2e–19
RNAseq + MRI T2-FLAIR	Joint MDS	46.7 ± 0.07	–	71.6 ± 0.12	–
	Baseline—Pamona	52.2	7.7e–7	66.7	3e–2
	Baseline—SCOTv2	54.1	8.5e–10	64.3	4e–5
Methylation + MRI T2-FLAIR	Joint MDS	47.5 ± 0.01	–	**72.1 **± 0.01	–
	Baseline—Pamona	51.1	5.9e–37	65.1	1.0e–38
	Baseline—SCOTv2	42.5	5.7e–43	58.1	7.3e–56
CNV + MRI T2-FLAIR	Joint MDS	**51.7 **± 0.02	–	55.5 ± 0.1	–
	Baseline—Pamona	52.0	2e–1	58.7	1e–2
	Baseline—SCOTv2	52.5	4e–3	54.5	3e–1

a Joint MDS performance (mean ± standard deviation) using FOSCTTM and label transfer accuracy. Baseline performances are shown in grey, with *P*-values from Wilcoxin tests comparing Joint MDS to each baseline indicated next to the corresponding metric. The best FOSCTTM and transfer accuracy scores for each modality pair are bolded.

The Joint MDS plots contain two points per patient corresponding to data from each of the two modalities. Joint MDS on different combinations of sequencing modalities maintains the separation of GBM versus LGG patients (Fig. 3A–C), and outperforms both baseline methods in the FOSCTTM (alignment) and label transfer accuracy scores. Furthermore, Joint MDS of CNV with RNA-seq and with DNA methylation (Fig. 3B and C) visually show better segregation of the GBM patients which was not evident in the CNV modality alone (Fig. 2A). Joint MDS applied to MRI radiomic features with all three sequencing modalities resulted in a poorer separation of GBM and LGG patients in contrast to the sequencing-only combinations. However, this approach still improved separation of GBM and LGG patients compared to that of the imaging modality alone (Fig. 2B). Interestingly, integration of radiomic features with RNA-seq revealed a small subcluster of MRI LGG patients amongst majority of GBM patients (Fig. 3D—left of the plot). Higher FOSCTTM scores when integrating sequencing and imaging modalities as compared to integrating sequencing-only modalities reflects the difficulty in aligning heterogeneous data modalities. Still, Joint MDS outperforms the baselines reflected by its lower FOSCTTM score (Table 1).

### 3.3 Application of joint MDS3

**Figure 4.**
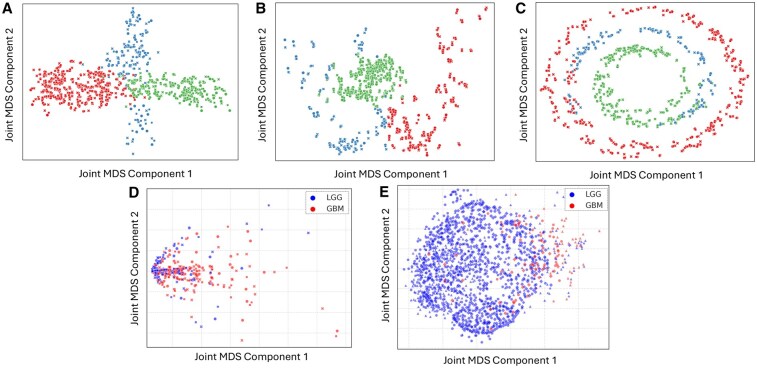
(A–C) Visualization of the results from the Joint MDS3 to align the triplets for each synthetic dataset Bifurcation (A), Swiss roll (B) and Circular Frustrum (C), coloured by their labels. (D) Visualization of the results from Joint MDS3 applied to align three different MRI contrast images, T1-contrast enhanced (T1C), T2-FLAIR, and T2-weighted (T2W). (E) Visualization of the results from Joint MDS3 applied to align three different sequencing modalities (RNA-seq, DNA methylation and copy number variants), with GBM patients are coloured in red and LGG patients are coloured in blue.

To demonstrate our extended Joint MDS for three domains, we tested the algorithm on three (triplets of) synthetic datasets. Figure 4A–C shows the joint visualizations of the aligned triplets for each synthetic dataset with their performance evaluation scores in Table 2. Joint MDS3 successfully aligned the three data domains for each synthetic dataset with low FOSCTTM scores of less than 10% and high accuracy scores of 95% and over.

**Table 2.** Results of Joint MDS3 performance.^a^

Modality category	Domains	FOSCTTM	Transfer accuracy
Synthetic data	Bifurcation	7.3	95.7
	Swiss Roll	1.0	96.3
	Circular Frustrum	0.4	95.3
Imaging data	MRI images (T1, T2F, T2W)	8.7	68.3
Omics data	RNASeq, CNV, DNA Methylation	29.2	93.2

a Joint MDS3 performance evaluated using average fraction of samples closer than the true match (FOSCTTM) score and the label transfer accuracy score.

### 3.4 Joint MDS3 on real-world datasets

Joint MDS3 was tested on two real-world triplets, radiomic features from three different MRI contrasts and sequencing (RNA-seq, DNA methylation, CNV). The resulting joint visualization plots are shown in Fig. 4D and E, and performance evaluation scores are presented in Table 2 (see Supplementary Appendix S2.4 for runtime evaluation). Joint MDS3 managed to align the three MRI contrasts, T1C, T2-FLAIR, and T2W (Fig. 4D) as well as the three sequencing data modalities (Fig. 4E) while maintaining the separation of GBM patients from LGG patients. However, the alignment of the imaging domains appears to be better, reflected by the lower FOSCTTM compared to that of the three sequencing data modalities.

Both imaging and molecular characterization are essential for comprehensive understanding of cancer biology. Using brain tumours as a proof of principle application, we have shown that Joint MDS applied to six different modality pairs outperforms baselines Pamona in all six cases, and SCOTv2 in four out of six cases (Table 1). For the two exceptions, SCOTv2 achieves slightly better performance: in the CNV–methylation pair, SCOTv2 outperforms JMDS in both FOSCTTM and label transfer accuracy, while in the methylation–MRI pair, SCOTv2 outperforms JMDS only in FOSCTTM, with JMDS retaining superior transfer accuracy. These results highlight the robustness and competitive performance of JMDS, particularly relative to Pamona. Our results also show the effectiveness of integrating sequencing data with radiomic features to improve the separation of GBM and LGG patients. This is particularly evident in the enhanced visual separation of GBM and LGG patients upon integration of MRI T2-FLAIR modality with sequencing data (Fig. 3D–F). Juxtaposing the contrasting views of sequencing and radiomic features reveals nuanced insights that are not apparent when using imaging alone (Fig. 2B), highlighting the complementary role of sequencing data in refining patient separation. Notably, in Fig. 3D, the integration of radiomic features with RNA-seq data revealed a small subcluster of MRI LGG patients within the GBM cluster. A Mann–Whitney U test showed no significant difference between this subcluster and the surrounding MRI GBM patients, but a significant difference was observed relative to the larger MRI LGG cluster (Fig. 3D). While this subcluster could potentially represent patients transitioning to a higher-grade tumour, these RNA-seq labels reflect bulk sequencing and may be limited in capturing tumour heterogeneity, whereas MRIs could provide additional context. Given the modest sample size, these observations should be interpreted cautiously, and further validation by radiology and oncology experts is needed. Nonetheless, this approach demonstrates the potential of multi-modal integration to highlight candidate patient subgroups for further study.

We have also managed to successfully extend Joint MDS to align data from three domains, demonstrated on the synthetic datasets. For the real-world datasets, we focus on applying Joint MDS3 to ‘similar’ modalities first as a means of testing its applicability to a simpler problem. This approach allows for a gradual exploration of the algorithm’s capabilities before tackling the integration of more heterogeneous modalities. Although the three imaging contrasts were successfully aligned with a low FOSCTTM score (Table 2), interpreting the separation of LGG and GBM patients (Fig. 4D) is challenging, as it reveals an indistinct separation with GBM patients exhibiting a more dispersed distribution, reminiscent of the individual modality results (Fig. 2B). This dispersion among GBM patients may mirror the less defined borders typically observed in MRIs of GBM patients (Malik *et al.* 2021). Additionally, using PyRadiomics for feature extraction could contribute to this pattern, as the extracted features lack clear biological interpretations.

These results highlight the potential of Joint MDS and its extension towards enhancing our understanding of complex disease mechanisms. By revealing new subclusters and relationships between different data modalities, Joint MDS offers a powerful tool for unravelling the complexities of various diseases, including cancers and other multifaceted conditions. This holistic approach to multi-modal data integration can provide valuable insights into biomarker discovery and treatment response prediction across a wide range of medical fields.

Although integrating heterogeneous modalities captures different aspects of disease biology, the limitation of low sample sizes is unavoidable when working with real-world clinical data. Only a small subset of TCGA patients have both imaging and multi-omics data available, reflecting the broader challenge of collecting comprehensive multi-modal datasets. However, our goal was to benchmark existing unsupervised manifold alignment techniques, showing that Joint MDS is a feasible and effective approach, while acknowledging that larger cohorts will be needed to assess generalizability. Furthermore, exploring advanced methods like neural network-based feature extraction or transfer learning using pre-trained networks may be interesting alternatives to process imaging data (Puls *et al.* 2024). Neural networks can automatically learn discriminative features from raw data, while transfer learning leverages pre-trained networks to improve performance on smaller, domain-specific tasks (Zhuang *et al.* 2020). Features derived from convolutional network architectures could improve the discriminative power of radiomic features to better distinguish between LGG and GBM patients compared to pre-extracted features using PyRadiomics, aiding in effective analysis of complex biological diseases like cancer. A technical limitation of Joint MDS and Joint MDS3 is the requirement for patient data to be available for all modalities of interest. Future work could address this by using clustering algorithms to find similar patients with complete modality information and applying their transportation cost values to interpolate patients with missing modalities. Scalability and computational efficiency are important considerations for multi-modal data integration algorithms like Joint MDS. Joint MDS3 has quadratic computational complexity in the number of samples per modality (see Supplementary Appendix S2.5), which is manageable for typical multi-modal oncology datasets but may require GPU acceleration or further optimization for very large cohorts. Future approaches, such as multi-marginal optimal transport, could improve runtime and flexibility for aligning more than three domains.

Additionally, using spatial sequencing data, such as spatial transcriptomics, could also enhance multi-modal integration by mitigating potential sampling biases, providing a more comprehensive understanding of tumour biology that accounts for spatial heterogeneity. These avenues hold promise for extending the utility of Joint MDS across diverse biomedical applications and offer new opportunities to uncover underlying patterns, ultimately contributing to the advancement of personalized medicine.

## Supplementary Material

btaf597_Supplementary_Data

## Data Availability

Sequencing data was obtained from The Cancer Genome Atlas (TCGA) and MRIs were obtained from the 2023 Brain Tumour Segmentation Chal-lenge (Bran Li *et al*. 2023).
