# Supplementary material for: Joint representation learning for oncology applications
Source: Bioinformatics. 2025 Oct 29;42(1):btaf597. doi: 10.1093/bioinformatics/btaf597 (PMC12790816; doi:10.1093/bioinformatics/btaf597)

Appendix

# **A. Hyperparameter Grid Search**

We conducted an extensive grid search to select optimal hyperparameters for the Joint Multidimensional Scaling (Joint MDS) algorithm. The grid search involved systematically evaluating various combinations of hyperparameters to identify the optimal settings for aligning different modality pairs. The hyperparameters considered include:

1. k-nearest neighbours ($k$): nearest neighbours to compute geodesic distances; k ranging from 3 to 10 were explored.
2. Entropic regularisation ($\epsilon$): this term in the Wasserstein-Procrustes analysis adds stability to the optimization process by penalizing large transportation costs. Values of $\epsilon$ ranging from 0.1 to 0.5 were tested.
3. Matching penalty (λ): this parameter controls the importance of matching correspondences between data instances from different domains. Higher values of λ prioritize accurate alignment at the expense of increased computational complexity. We tested ε values ranging from 0.1 to 1.
4. Mode: This parameter specifies the mode used for computing pairwise dissimilarities. In Joint MDS, two modes are commonly used:
   1. Connectivity: In this mode, pairwise dissimilarities are computed based on the connectivity between data points. It considers the presence or absence of connections between data points in a graph representation.
   2. Distance: pairwise dissimilarities are computed based on the Euclidean distance or other distance metrics between data points in the high-dimensional space and it directly measures the distance between data points without considering their connectivity.
5. Metric: This parameter specifies the distance metric used for computing pairwise dissimilarities when the mode is set to distance. Common distance metrics include:
   1. Correlation: Calculates the correlation coefficient between data points, measuring the linear relationship between variables
   2. Minkowski: Computes the Minkowski distance between data points, which is a generalization of other distance metrics such as Euclidean distance (when the parameter p=2) and Manhattan distance (when p=1).

The grid search involved systematically evaluating the performance of Joint MDS for each combination of hyperparameters on a validation dataset. Performance metrics such as the fraction of samples closer than the true match (FOSCTTM) and label transfer accuracy were used to assess the alignment quality.

# **A.1. RNA-seq and DNA Methylation (Joint MDS)**

For this pairwise combination, $\varepsilon=0.01$ and $\lambda=0.1$ for a maximum number of 500 iterations.

For RNA-seq: $k=30, metric=minkowski, mode=distance$

For DNA Methylation: $k=50, metric=minkowski, mode=distance$

# **A.2. RNA-seq and CNV (Joint MDS)**

For this pairwise combination, $\epsilon=0.01$ and $\lambda=0.1$ for a maximum number of 500 iterations.

For RNA-seq: $k=30, metric=minkowski, mode=connectivity$

For CNV: $k=60, metric=correlation, mode=connectivity$

# **A.3. DNA Methylation and CNV (Joint MDS)**

For this pairwise combination, $\epsilon=0.01$ and $\lambda=0.1$ for a maximum number of 500 iterations.

For DNA Methylation: $k=20, metric=correlation, mode=connectivity$

For CNV: $k=20, metric=correlation, mode=connectivity$

# **A.4. RNA-seq and MRI T2-FLAIR (Joint MDS)**

For this pairwise combination, $\epsilon=0.5$ and $\lambda=0.5$ for a maximum number of 500 iterations.

For MRI T2-FLAIR: $k=60, metric=correlation, mode=distance$

For RNA-seq : $k=30, metric=mikowski, mode=connectivity$

# **A.5. DNA Methylation and MRI T2-FLAIR (Joint MDS)**

For this pairwise combination, $\epsilon=0.01$ and $\lambda=0.1$ for a maximum number of 500 iterations.

For MRI T2-FLAIR: $k=30, metric=mikowski, mode=distance$

For DNA Methylation: $k=50, metric=minkowski, mode=distance$

# **A.6. CNV and MRI T2-FLAIR (Joint MDS)**

For this pairwise combination, $\epsilon=0.1$ and $\lambda=0.5$ for a maximum number of 500 iterations.

For MRI T2-FLAIR: $k=20, metric=correlation, mode=distance$

For CNV: $k=60, metric=correlation, mode=distance$

# **A.7. Hyperparameters for Baselines Pamona & SCOTv2**

For SCOTv2, parameters are kept the same as outlined in the original implementation

- k=50
- 𝜀=0.005
- ε=0.005
- ρ=0.1
- projMethod="barycentric"

For Pamona, the parameters are

- λ=10 (fixed)
- n_shared = number of shared samples, which depends on the specific modality pair, as shown in the table below


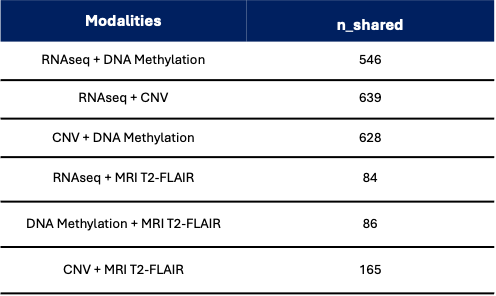


# **B. Experimental details and additional information**

# **B.1. Computational details**

All experiments were run on the ETH Euler cluster with 30 cores and 64 GB of RAM.

# **B.2. Runtime of Joint MDS**

Implementations of Joint MDS on combinations of sequencing-only modalities took an average of 307 seconds, while implementations of Joint MDS on combinations of imaging too an average of 66 seconds due to the low number of shared patients. Exact runtimes for each pairwise implementation are shown below:

# **B.3. Performance Evaluation of Joint MDS**

To provide a more detailed view of JMDS performance, Supplementary Table S1 reports the fraction of runs in which JMDS outperformed each baseline (Pamona and SCOTv2) across 50 independent runs with different random seeds. For each modality pair and metric (FOSCTTM and label transfer accuracy), we counted the number of JMDS runs that achieved a better score than the corresponding baseline and divided by the total number of runs, yielding a fraction between 0 and 1.

This analysis complements the main results (Table 1) by illustrating the robustness and consistency of JMDS across multiple stochastic realizations. Values close to 1 indicate that JMDS consistently outperforms the baseline, while values near 0.5 indicate comparable performance. All baselines were implemented according to the original publications, and metrics were calculated as described in the Methods section.


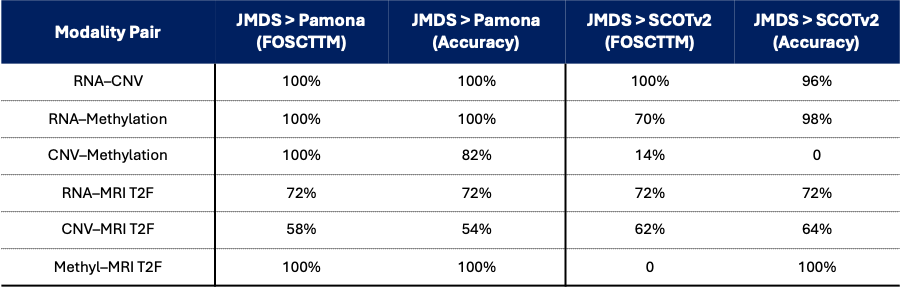


# **B.4. Runtime of Joint MDS3**

Aligning the synthetic datasets took an average of 62 seconds. The real-world dataset runtimes were longer, given the more complex nature of the data and that they are less similar to each other than the triplets within each synthetic dataset. Exact runtimes for each triplet implementation are shown below:

# **B.5. Computational Complexity & Scalability**

JointMDS3 aligns multiple modalities by optimizing a coupling over intra-domain dissimilarities and jointly embedding samples. The dominant computational costs arise from:

1. Computing intra-domain pairwise distances.
2. Solving the entropic Gromov-Wasserstein (GW) problem.
3. Performing classical multidimensional scaling (MDS) for joint embedding.

For a pairwise alignment of two modalities:

$$O\left( T\cdot N^{2}\cdot d^{3} \right)$$

where 𝑇 is the number of iterations, 𝑁 the number of samples in each domain, and 𝑑 the embedding dimension

For JMDS3 (three modalities), the total complexity is approximately the sum of the three pairwise alignments. The quadratic term in N dominates; the cubic term in 𝑑 is negligible as 𝑑 is small (typically 2–3). Memory usage is primarily driven by the storage of distance matrices and GW coupling matrices for each pairwise alignment.

We measured wall-clock runtimes for cohorts of N = 25–150 patients on multi-omics datasets, averaged over multiple seeds (mean ± SD). Runtime scales approximately quadratically with sample size, consistent with theoretical predictions.


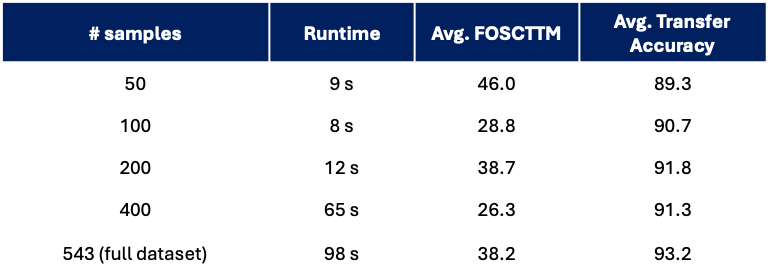

Supplement: btaf597_Supplementary_Data [file btaf597_supplementary_data.docx]
